# Food advertising on YouTube channels aimed at children in Brazil

**DOI:** 10.11606/s1518-8787.2023057004174

**Published:** 2023-07-31

**Authors:** Juliana de Paula Matos, Polyanna Botrel Tobias, Lavínia Baldim, Paula Martins Horta

**Affiliations:** I Universidade Federal de Minas Gerais School of Medicine Graduate Program in Health Sciences Belo Horizonte MG Brazil Universidade Federal de Minas Gerais. School of Medicine. Graduate Program in Health Sciences. Belo Horizonte, MG, Brazil.; II Universidade Federal de Minas Gerais School of Nursing Department of Nutrition Belo Horizonte MG Brazil Universidade Federal de Minas Gerais. School of Nursing. Department of Nutrition. Belo Horizonte, MG, Brazil.

**Keywords:** Food Advertising, Social Media, Child, Industrialized Foods

## Abstract

**OBJECTIVE:**

To analyze food advertising on YouTube channels aimed at children in Brazil and the interaction of the public with this type of advertising.

**METHODS:**

We analyzed the 10 most popular videos from the 25 YouTube most-watched channels with content aimed at children in the country in 2018. The presence of general advertising, food brands and food services was identified. When there was advertising in the videos, the foods and their respective brands were described, the first being classified according to the NOVA system. In cases of advertising of a specific food brand without its product having been displayed or mentioned, the classification was carried out according to the predominance of that company products. The number of visualizations and interactions (“likes” and “dislikes”) was also collected.

**RESULTS:**

General advertising was identified in 45.6% of videos, while food and food service advertising was present in 12.9% and 1.6% of videos, respectively. Food advertisements were mostly represented by ultra-processed products (n = 30; 93.8%). In channels led by *Kid YouTubers,* there was a higher frequency of general advertising, food and food services in the videos. In these channels, the advertisements of food in general and ultra-processed foods were respectively 2.79 and 2.53 times higher than in videos of channels not led by *Kid YouTubers* . The number of times videos were tagged “liked” was higher in videos with food advertising (1.67 × 10^5^) compared to videos without food advertising (1.02 × 10^5^), p = 0.0272.

**CONCLUSION:**

YouTube is a potential medium for children’s exposure and interaction with ultra-processed food advertising. The results of this analysis reinforce the importance of enforcing regulations prohibiting children’s advertising on this platform.

## INTRODUCTION

The participation of ultra-processed foods in the diet of Brazilians is 20% of the calories consumed, while *fresh* or minimally processed foods, which should be the basis of intake^1^, represent just over half (53.4%) of the calories consumed^2^. This scenario is related to the recent processes of urbanization, industrialization and modernization, which led to the development of a food environment led by large ultra-processed food companies^3^.

The dietary pattern with a high participation of ultra-processed foods is associated with excessive weight gain in children^4^. In Brazil, data from the Food and Nutrition Surveillance System (Sisvan) from 2020 show that 15.8% of children between 5 and 10 years old are overweight and 9.4% are obese^5^. Childhood obesity implies high costs for the health system, arising from the medication and treatment of diseases related to excess weight, in addition to possibly resulting in less productive adults with poorer quality of life^6^.

Excessive exposure to advertising of unhealthy foods is one of the determinants of childhood obesity^7^, since it can influence children’s food preferences, favoring the consumption of the advertised foods. This is due to the difficulty of children in recognizing the marketing character of advertising campaigns, given their cognitive limitations^8^. For this reason, health organizations consider advertising aimed at children an inappropriate and unfair practice, which needs to be regulated^9^.

In Brazil, all advertising directed at children is considered abusive and illegal by the Consumer Protection Code (CDC)^10^ and Resolution No. 163/2014 of the National Council for the Rights of Children and Adolescents (Conanda)^11^. However, food advertising monitoring data showed that half of the ads of this type served in 2018, in the programming of three channels open on Brazilian television, had content aimed at children with messages encouraging the excessive consumption of ultra-processed foods^12^. In addition, a recent study monitoring the official pages of companies producing this type of food on social media platforms showed the use of advertising strategies aimed at children, such as the presence of children’s characters and the distribution of gifts or collectibles, especially on YouTube^13^.

In the digital environment, YouTube is a video sharing platform used for different types of communication, including advertising^14^. Children have been active users of this platform, both as viewers and sharing their own content ( *kid youtubers)* . Although YouTube has registration restrictions for individuals under 13, it is possible to access the content of the platform without the need to register. In addition, there is a specific content platform for children, YouTube Kids, which restricts the advertising of paid ads that promote food, regardless of their nutritional content, however, there is no prohibition on the content of videos uploaded by users who have an active account on the platform, and it is possible that advertising practices not subject to the platform’s advertising policies are present in YouTube Kids videos^15^.

Currently, the advertising content produced by ultra-processed food companies on YouTube channels in Brazil is known^13^, but the presence of food advertising on YouTube channels aimed at children is yet unexplored. This platform is popular among Brazilian children and, according to data from media monitoring surveys, 78% of a sample (n = 1,628) of parents of children between 10 and 12 years old say that their children access YouTube on their *smartphones* ^16^. Added to this is the high amount of YouTube’s global advertising revenues, which, in 2020 alone, totaled US $19.77 billion^17^.

In the digital environment, children can interact directly with advertising content through likes, comments and shares, as well as access online links and games^18^. Thus, it is expected that the impact of children’s exposure to food advertising on their eating behavior will be even more relevant in this medium than in traditional media^19^. Therefore, this article aimed to analyze the advertising of food on YouTube channels aimed at children in Brazil in 2018 and the interaction of the public with this advertising.

## METHODS

The sample of YouTube channels used in the study was selected from the list of the 250 most viewed channels in the country, regardless of the target audience or subject covered, according to SocialBlade – YouTube’s independent social media analytics website^a^ – on November 23, 2018. Each channel on this list was analyzed individually, according to its description and indicated target audience, and the first 25 channels that presented content aimed at children were of interest to the study ( Chart 1 ). For this, the criteria of Conanda Resolution No. 163/2014^11^ were adopted, which qualifies the elements of a marketing communication directed to the child, such as the presence of: child language, special effects and excess of colors; soundtracks of children’s music or sung by children’s voices; representation of children (for example, *kid youtubers)* ; people or celebrities with appeal to children’s audiences; children’s characters or presenters; cartoons or animation, and dolls or the like.

**Chart 1:** Top 25 YouTube channels of children’s content; sorted by total number of views.

Channel name	Starting date	Ranking	Subscribers	Views
Galinha Pintadinha	08/30/2006	2	14,313,531	10,169,800,278
Turma da Mônica	07/30/2012	4	9,328,729	7,267,014,998
Totoykids	12/02/2014	8	11,682,586	5,683,096,765
Erlaina and Valentina	04/13/2014	28	10,411,610	2,534,875,335
PatatiPatatá	08/15/2011	2	3,756,167	2,528,137,084
Paulinho and Toquinho	11/03/2015	3	4,294,953	2,381,444,545
Canal Kids HD	05/02/2017	32	5,524,762	2,328,708,524
Planeta das Gêmeas	11/20/2015	36	9,056,517	2,175,707,183
Fran, Nina e Bel para meninas	08/10/2013	39	6,663,106	1,942,418,375
Julia Minegirl	09/21/2015	41	3,435,392	1,924,067,618
O Reino das Crianças	08/19/2014	42	3,165,953	1,903,409,294
KidsFun	08/26/2015	43	8,528,323	1,854,220,383
TOYSBR Parque dos Brinquedos Surpresa Brasil	11/06/2013	44	3,599,308	1,854,989,235
Chiquititas SBT	05/22/2013	45	3,547,862	1,811,340,664
CartoonKIDS BR	10/21/2017	48	4,693,485	1,787,588,001
Bela Bagunça	03/27/2015	52	8,009,492	1,628,998,411
Juliana Baltar	05/09/2010	58	7,701,365	1,527,991,011
Maria Carla e JP	09/05/15	59	5,784,087	1,507,850,538
Clubinho da Laura	08/19/12	62	5,167,755	1,475,654,566
Xuxa VEVO	09/11/09	71	1,183,641	1,337,921,235
Fran para meninas	09/23/12	80	4,891,034	1,184,063,769
CanalKids- Tatá e Henrique	7/21/15	81	3,743,958	1,152,844,510
Canal da Lelê	08/25/14	85	3,819,222	1,125,696,017
Disney Channel Brazil	05/03/08	86	2,560,522	1,109,790,068
3 Palavrinhas	09/24/13	96	1,381,620	970,358,193

The 10 most popular videos from each of the 25 selected channels were included in the study (n = 250). This number was established based on the methodology of a similar study carried out in Malaysia^20^. At the time of data collection, two videos were not available for viewing due to content deletion, totaling a final sample of 248 videos.

All videos were watched independently by two researchers on the YouTube platform, using the Google Chrome browser, from January to February 2019. The programming of the videos totaled 33 hours and 39 minutes. The date of publication of the videos as well as the data on the number of views and interactions (“liked” and “disliked”) were recorded and tabulated separately by the researchers in Excel spreadsheets. Besides, the researchers identified whether the channel was led by *Kid YouTubers* or not.

For the collection of advertising data in videos, it was considered there are four main types of ads on YouTube: (a) non-mandatory viewing ads or videos, that is, it is possible to proceed to the video of interest without viewing them, (b) mandatory viewing ads or videos that, regardless of their duration, do not make it possible to access the video of interest without first viewing them, (c) ads in stripe format that appear at the top or bottom of the video and (d) brands and products mentioned or shown in the scenario or by the characters/presenters of the videos. The first three types of ads, (a), (b) and (c), may appear at any time in the video, when authorized by the channel owner, who chooses the format and frequency, but not the content of the advertising. These ads were excluded from this study because they did not present a display pattern, having no relation to the content of the videos. Thus, only the brands mentioned or shown by videos and that fall into type (d) were considered as advertising. In this case, the publicity was given by the representation of the child consuming the brand’s product, by the demonstration of characteristics of the brand’s food or by the display of the food brand in the background of the video ( Table 2 ).

**Table 2:** Food companies and food services in the advertising of YouTube videos aimed at children in Brazil, 2018.

Company Segment	n	Company (number of brands)^a^
/minimally Processed foods	2	Yoki (1); Fina (1)
Culinary Ingredients	1	Liza (1)
Ultra-processed	45	
Sweets and treats	18	Mondelez (4); Nestlé (3); Ferrero (3); Chupa-Chups (1); Perfetti Van Melle (1); Garoto (1); Dori (1); Haribbo (1); Fini (1); Ovomaltine (1); Mars (1)
Dairy drinks	6	Danone (2); Nestlé (2); Sufresh (1); Pepsico (1)
Bundled Breads & Biscuits	9	Mondelez (2); Nestlé (2); Pandurata Alimentos (1); Parati (1); Pepsico (1); Richester (1); Wickbold (1)
Juices and soft drinks	5	Coca-Cola (3); Pepsico (1); Active Plus (1)
	5	Pepsico (5)
Ultra-processed meats	1	BRF (1)
Breakfast Cereals	1	Kelloggs (1)
Food services	4	McDonalds (1); Burger King (1); Hot Box (1); Los Paleteros (1)
Total	52	

^a^ The same company may be common to more than one brand in the sample.

All advertisements identified in the videos were classified according to the type of product advertised in: general advertising, such as toys and games, and food advertising and food services. When the presence of food advertising was identified in the content of the video, a description of the products and their respective brands was made. These first were grouped into *fresh* and minimally processed foods, culinary ingredients, processed and ultra-processed foods, according to the NOVA classification proposed by Monteiro et al.^21^ In the case of ultra-processed food advertisements, the products were classified into different groups, namely: sweets and treats, dairy drinks, juices and soft drinks, bread and biscuits, *chips* , ultra-processed meats, and breakfast cereals. In cases where there was a display or mention of a food brand in the video, without its product being displayed or mentioned, food advertising was considered and the classification was carried out according to the predominance of the products of that company. For this, the product portfolio was analyzed on the companies’ official websites. In all cases, the companies corresponding to the brands found in the videos were identified.

At the end, data sheets prepared by the study researchers were compared and all inconsistencies corrected, to ensure greater data reliability, whose analysis included the description of the videos regarding the number of views and “like”- and “dislike”-type interactions. For this, we used the median and the interquartile range (25th percentile; 75th percentile), because the distribution of these variables did not adhere to the normal distribution curve, according to the Shapiro-Wilk normality test. In addition, the frequency of general advertising, food (and its subclassifications) and food services was calculated, as well as the number of food advertising placements in each video. This frequency was estimated in the general sample and in the videos of channels led or not by *Kid YouTubers* . Finally, the medians of the number of views and “liked”- and “disliked”-type interactions were compared between the videos that had general advertising, food and food services, through the application of the Mann-Whitney test, at a significance level of 5% (p < 0.05). Analyses were performed using the statistical software Stata, version 12.0.

**Chart 2:** Screenshots with examples of food advertising in YouTube videos directed to children in Brazil, 2018.

Ad Type	Context of appearance of food
Representation of individual consuming the brand product	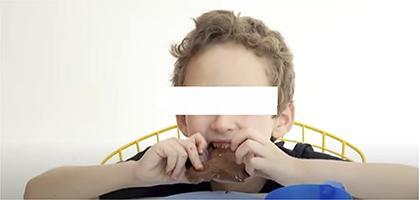
	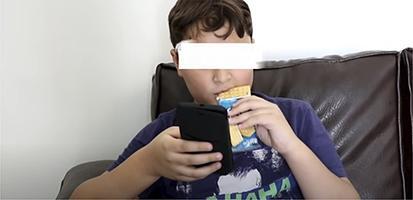
Demonstration of brand food characteristics	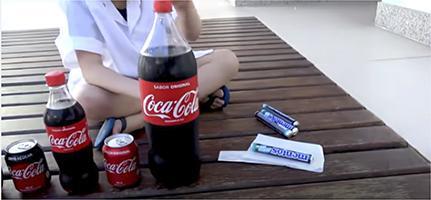
	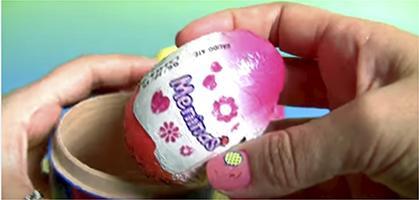
Display of the food brand in the background	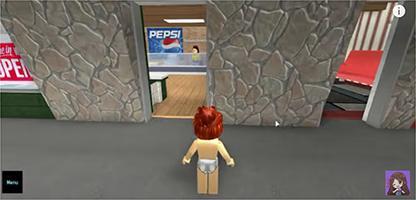
	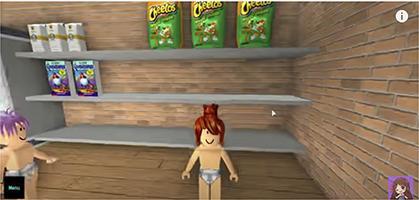

## RESULTS

The sample consisted of videos published between 2006 and 2018, with the majority (83.2%) posted between 2015 and 2018. The videos had a large number of views (median: 2.93 × 10^7^) and interactions (median “liked”: 1.09 × 10^5^; median “disliked”: 0.25 × 10^5^). Of the total channels analyzed, 9 (36%) were from *kid youtubers* (data not shown in the table).

General advertising was identified in 45.6% (n = 113) of videos, while food and food service advertising was in 12.9% (n = 32) and 1.6% (n = 4) of the videos, respectively. Food advertisements were mostly represented by ultra-processed foods (n = 30; 93.8%) and, among these, by sweets and treats (n = 21; 70%). In channel videos led by *kid youtubers,* there was a higher frequency of overall advertising (75.6% vs. 30.7%), food (24.4% vs. 7.2%) and food services (3.7% vs. 0.6%). In addition, ads for the ultra-processed food subgroup were more frequent in videos led by *kid youtubers* , except for ads for juices or soft drinks and breakfast cereals – videos led by *kid youtubers* : 16.7% and 0%, respectively, and videos not led by *kid youtubers* 25% and 8.3%, respectively ( Table 1 ).

**Table 1:** Characterization of advertising present in YouTube videos directed at children in Brazil, 2018.

Ad Type	Presence of advertising^a^						Food advertising placements^b^		
	
	Overall sample		Channel videos of *kid youtubers*				Overall sample	Channel videos of *kid youtubers*	
	
			Yes		No			Yes	No
					
	n	%	n	%	n	%	n	n	n
General (except food)	113	45.6	62	75.6	5	30.7	-	-	-
Food	32	12.9	20	24.4	12	7.2	72	5	19
/minimally Processed foods	2	6.3	2	1	0	0	3	3	0
Culinary Ingredients	2	6.3	2	1	0	0	2	2	0
Ultra-processed	30	93.8	18	90	12	100	67	48	19
Sweets and treats	21	70	14	77.8	7	58.3	30	23	7
Dairy drinks	7	23.3	5	27.8	2	16.7	8	6	2
Bundled Breads & Biscuits	5	16.7	4	22.2	1	8.3	1	6	4
Juices and soft drinks	6	20	3	16.7	3	25	1	6	4
	3	1	2	11.1	1	8.3	7	6	1
Ultra-processed meats	1	3.3	1	5.6	0	0	1	1	0
Breakfast Cereals	1	3.3	0	0	1	8.3	1	0	1
Food services	4	1.6	3	3.7	1	0.6	4	3	1

^a^Refers to the presence of advertising (Yes or No). The same advertisement may contain general, food and food service advertising at the same time. Similarly, an advertisement may have more than one type of food being advertised.

^b^Refers to the total number of times that advertising for a particular type of product was noticed in the videos evaluated. Applies only to food advertising and food services.

In addition, of the 30 videos that contained advertising of ultra-processed foods, 67 ad placements were counted. Advertisements for sweets and treats (n = 30), juices and soft drinks (n = 10) and packaged breads and cookies (n = 10) were the most frequent incidents. In the case of channel videos led by *kid YouTubers* , food advertising placements were 2.79 times more frequent than in channel videos not led by *kid YouTubers* . For the advertising of ultra-processed foods, this ratio was 2.53 times ( Table 1 ).

As for the brands evidenced/mentioned in the videos, there was greater participation of transnational companies in the candy and treats segment (18 different brands) and producers of breads and cookies (nine brands). The most frequent brands are from transnational companies such as Pepsico, Mondelez and Nestlé ( Table 2 ).

The number of views of videos with general advertising was lower than those without it: 2.60 × 10^7^ vs. 3.41 × 10^7^, p = 0.0391. The number of “likes” was higher among videos with general advertising (1.35 × 10^5^) compared to videos without general advertising (0.84 × 10^5^), p = 0.0001 ( Table 3 ).

**Table 3:** Association between the presence of general, food, and food services advertising in YouTube videos aimed at children in Brazil with the number of views and user engagement, 2018.

Ad Type	Views		“Liked”		“Disliked”	
		
	Median	P25; P75	Median	P25; P75	Median	P25; P75
General						
Yes	2.60 × 10^7^	1.74 × 10^7^; 3.93 × 10^7^	1.35 × 10^5^	0.88 × 10^5^; 2.50 × 10^5^	0.29 × 10^5^	0.16 × 10^5^; 0.46 × 10^5^
No	3.41 × 10^7^	1.47 × 10^7^; 7.30 × 10^7^	0.84 × 10^5^	0.54 × 10^5^; 1.75 × 10^5^	0.23 × 10^5^	0.14 × 10^5^; 0.47 × 10^5^
P value	0.0391		0.0001		0.2032	
Food						
Yes	2.55 × 10^7^	1.75 × 10^7^; 3.92 × 10^7^	1.67 × 10^5^	1.04 × 10^5^; 3.27 × 10^5^	0.33 × 10^5^	0.23 × 10^5^; 0.39 × 10^5^
No	3.07 × 10^7^	1.53 × 10^7^; 5.97 × 10^7^	1.02 x 10^5^	0.59 × 10^5^; 1.96 × 10^5^	0.24 × 10^5^	0.13 × 10^5^; 0.52 × 10^5^
P value	0.5938		0.0272		0.185	
Food services						
Yes	2.71 × 10^7^	2.57 × 10^7^; 3.84 × 10^7^	1.47 × 10^5^	1.37 × 10^5^; 1.97 × 10^5^	0.34 × 10^5^	0,33 × 10^5^; 0,35 × 10^5^
No	2.96 × 10^7^	1.57 × 10^7^; 5.75 × 10^7^	1.06 × 10^5^	0.59 × 10^5^; 2.15 × 10^5^	0.24 × 10^5^	0.14 × 10^5^; 0.51 × 10^5^
P value	0.9824		0.2259		0.346	

In the case of food advertising, likewise, the number of times videos were marked with “liked” was higher in the presence of this type of advertising (1.67 × 10^5^) compared to videos without food advertising (1.02 × 10^5^), p = 0.0272 ( Table 3 ).

## DISCUSSION

This study evidenced the fact that general and food advertising is significantly present in Brazilian YouTube channels aimed at children, especially in channels led by *kid youtubers* . In addition, it was identified that user interaction by marking “I liked” is greater when there are advertisements, regardless of the type of product being promoted.

These results denote the high potential of exposure of Brazilian children to advertising content on YouTube channels in Brazil, especially when it comes to ultra-processed foods from important transnational companies. This scenario is critical because these are advertising messages that target an admittedly vulnerable audience^9^,which should be protected from this type of message. This exhibition takes place on a medium (YouTube) of great popularity, which can be accessed by children at any time by different devices, without the direct supervision of their parents or guardians. Added to this is the well-known association between exposure to food advertising and preference for advertised products^8^ and between the consumption of ultra-processed foods and unfavorable health outcomes in children and adolescents, including obesity^4^.

The results of the study also show that, in addition to children being an audience for the published content, they are also creators of content, as in the case of *kid youtubers* . In the videos produced by these children, there was a higher frequency and higher number of food advertising placements. The content of *kid youtubers* videos contains subtle integrations between brand advertising and entertainment, which makes them more difficult to be recognized as advertising content^22^. In addition, when commercial content is endorsed by a child, it becomes an important influencer for the consumption decisions of its audience, which usually consists of other children^22^.

The rate of ultra-processed food advertising identified in this study is concerning. Similar results were found in Malaysia, where 56.3% of food advertising served on children’s YouTube channels was from ultra-processed foods, mainly from *fast food* and sweets, resulting in the transmission of 0.73 ads per hour of video^20^. In the United Kingdom, 92.9% of videos of children’s influencers on YouTube suggested some food or drink^23^, while in Spain this rate was 73.6%^14^.

In addition to the presence of food advertising on YouTube channels aimed at children in Brazil, an expressive interaction with the content of the videos was identified, in the presence of advertising content, by the marking “liked”. Data in the literature indicate that the greater the interaction of children with advertising, the greater the effect of exposure on eating behavior^19^. In Australia, for example, one study showed that 12- to 17-year-olds who liked or shared a food or drink post on social media at least once in the month prior to the survey had a high intake of unhealthy foods, and the more the teens interacted with the content, the higher the consumption^24^.

Taken together, the evidence found in this study characterizes the low effectiveness of measures to regulate advertising aimed at children in Brazil, which are considered abusive and prohibited by law^10 , 11^. It is therefore necessary to advance this agenda, which implies updating the regulatory terms in the context of digital advertising and ensuring the inspection and punishment for this type of advertising.

In the first case, one must add to the regulatory texts the clear definition of advertising in the digital environment, including that carried out by digital influencers (such as *kid youtubers* )^25^. Some regulatory texts that can be used as inspiration for the regulation of digital food advertising in Brazil is Law No. 30/2019, of Portugal, which introduces restrictions on the advertising of unhealthy foods on the internet, on websites or social media, as well as on mobile applications, when their content is intended for children under 16^26^. In Latin America, the Chilean government also restricts advertising of foods deemed unhealthy targeting children under 14 on websites^27^.

In addition, it is recommended to impose limits on the targeting of advertising through individual data algorithms^25^, as companies can currently extract user location and browsing history data from a digital device and use it to personalize advertising messages. This is an automated process, called programmatic media, used in the purchase and delivery of ads through algorithms to find and target advertising according to the interests of individuals. Thus, advertisers can identify where the child is and their preferred content. With this, companies can experiment with different commercial strategies aimed at the individual’s consumption profile and monitor the individual’s interaction with the ad, as well as conversion into acquisition^18^.

In the case of inspection, it is necessary to invest in technologies for the systematic monitoring of digital advertising, which capture the multidimensionality of this type of media and the different ways of advertising^19^. One path would be the adoption of predefined instruments for the identification of food advertising aimed at children on YouTube channels, including on channels led by child influencers, as provided for in the protocols recently launched by the regional office of the World Health Organization in Europe^b^. It is also necessary to overcome the challenges for the monitoring of advertising disseminated by programmatic media, not addressed in this study, in the types of advertising that precede or are broadcast throughout the videos, usually of mandatory viewing, but which do not have an equal display pattern for individuals.

Social media platforms can also contribute to ensuring the protection of children from exposure to commercial content. In the case of YouTube, as already mentioned, there is no restriction on children’s advertising, even if it is possible for children to access the platform. As for YouTube Kids, although there is a ban on food advertising by large companies, this measure does not extend to influencer-generated content and there is evidence of great inadequacy and negligence on the part of the platform in inspection^28^.

Likewise, the food industry can act responsibly and make a commitment not to communicate directly with children. In Brazil, the National Council for Advertising Self-Regulation (Conar), composed of advertising agencies and advertisers, does not recognize advertising aimed at children as illegal and presents a performance more aligned with market relations than focused on the protection of children in the face of the power of advertising practices^29^.

It is worth discussing the legal precedent that fined an ultra-processed food company in Brazil, Pandurata Alimentos, for targeting advertising to children. Despite not being a case of widespread advertising in the digital environment, the focus of this study, the Superior Court of Justice (STJ) recognized that the company used a promotional strategy to encourage the consumption of low nutritional quality foods by children and, thus, contradicted the guidelines on abuse of the CDC^30^. Expanding the number of legal precedents regarding abusive advertising and expanding the recognition that it is also abusive to hire *kid youtubers* for product advertising are necessary ways to ensure the protection of children against marketing communication.

As for the potential limits of the study, it is first indicated the absence of a pattern of display of mandatory, optional and banner ads, which allowed us to evaluate only a portion of food advertising in videos aimed at children. Thus, it is estimated that the exposure of children to advertising content is even greater on this platform. Secondly, the impossibility of knowing the origin of the interactions, that is, how many of them actually came from the child audience, is also a limitation.

Despite this, it was possible to conclude that there is a predominance of advertising of ultra-processed foods in videos of children’s YouTube channels in Brazil, especially in channels led by *kid YouTubers* , which reinforces the importance of actions to regulate food advertising on social media and provides evidence for future investigations aimed at understanding the impact of childhood exposure to food advertising and the profile of children’s food consumption.

## CONCLUSION

There was an expressive presence of food advertising from important transnationals, with a high frequency of placements on children’s channels on YouTube, especially on channels led by *kid youtubers* . In addition, there were more “liked”-type interactions in videos with general or food advertising.

## Footnotes

a Social Blade. Top 100 youtubers in Brazil sorted by SB rank [internet]. 2018 [quoted nov. 23, 2018]. Available from: https://socialblade.com/youtube/top/country/br

b World Health Organization. Europe. Monitoring of marketing of unhealthy products to children and adolescents:– protocols and templates. 2020 [quoted aug. 20, 2021]. Available from: https://www.euro.who.int/en/health-topics/disease-prevention/nutrition/activities/monitoring-of-marketing-of-unhealthy-products-to-children-and-adolescents-protocols-and-templates
