# Supplementary material for: Food advertising on YouTube channels aimed at children in Brazil
Source: Rev Saude Publica. 2023 Jul 31;57:50. doi: 10.11606/s1518-8787.2023057004174 (PMC10392772; doi:10.11606/s1518-8787.2023057004174)
Supplement: Translation

# Translation: Publicidade de alimentos em canais do YouTube direcionados à criança no Brasil

**Authors:** Juliana de Paula Matos, Polyanna Botrel Tobias, Lavínia Baldim, Paula Martins Horta

**Affiliations:** I Brazil Universidade Federal de Minas Gerais. School of Medicine. Graduate Program in Health Sciences. Belo Horizonte, MG, Brazil.; II Brazil Universidade Federal de Minas Gerais. School of Nursing. Department of Nutrition. Belo Horizonte, MG, Brazil.

**Keywords:** Publicidade de Alimentos, Mídias Sociais, Criança, Alimentos Industrializados

## Abstract

**OBJETIVO:**

Analisar a publicidade de alimentos em canais do YouTube direcionados à criança no Brasil e a interação do público com esse tipo de publicidade.

**MÉTODOS:**

Foram analisados os 10 vídeos mais populares dos 25 canais do YouTube com conteúdo dirigido ao público infantil mais assistidos no país em 2018. Identificou-se a presença de publicidade geral, de marcas de alimentos e de serviços de alimentação. Quando houve publicidade nos vídeos, os alimentos e suas respectivas marcas foram descritos, sendo os primeiros classificados segundo o sistema NOVA. Nos casos de publicidade de uma marca de alimentos específica, sem que o seu produto tenha sido exibido ou mencionado, a classificação foi realizada segundo a predominância dos produtos dessa empresa. Coletou-se, também, o número de visualizações e de interações (“gostei” e “não gostei”).

**RESULTADOS:**

A publicidade geral foi identificada em 45,6% dos vídeos, enquanto a publicidade de alimentos e de serviços de alimentação esteve presente em 12,9% e 1,6% dos vídeos, respectivamente. Os anúncios de alimentos foram representados em sua maioria por produtos ultraprocessados (n = 30; 93,8%). Em vídeos de canais liderados por *youtubers mirins,* observou-se uma maior frequência de publicidade geral, de alimentos e de serviços de alimentação. Nesses canais, as veiculações de publicidade de alimentos em geral e de alimentos ultraprocessados foram respectivamente 2,79 e 2,53 vezes maior do que nos vídeos de canais não liderados por *youtubers mirins* . O número de vezes em que os vídeos foram marcados com “gostei” foi maior nos vídeos com publicidade de alimentos (1,67 × 10^5^) em comparação aos vídeos sem publicidade de alimentos (1,02 × 10^5^), p = 0,0272.

**CONCLUSÃO:**

O YouTube é um potencial meio de exposição e interação de crianças com a publicidade de alimentos ultraprocessados. Os resultados desta análise reforçam a importância de fazer cumprir a regulamentação de proibição de publicidade infantil nessa plataforma.

## INTRODUÇÃO

A participação de alimentos ultraprocessados na dieta dos brasileiros é de 20% das calorias consumidas, ao passo que os alimentos *in natura* ou minimamente processados, que deveriam ser a base da alimentação^1^, representam pouco mais da metade (53,4%) das calorias consumidas^2^. Esse cenário está relacionado aos recentes processos de urbanização, industrialização e modernização, que propiciaram o desenvolvimento de um ambiente alimentar protagonizado por grandes empresas de alimentos ultraprocessados^3^.

O padrão alimentar com alta participação de alimentos ultraprocessados está associado ao ganho de peso excessivo em crianças^4^. No Brasil, dados do Sistema de Vigilância Alimentar e Nutricional (Sisvan) de 2020 mostram que 15,8% das crianças entre 5 e 10 anos estão com sobrepeso e 9,4% com obesidade^5^. A obesidade infantil implica elevados custos para o sistema de saúde, advindos da medicação e tratamento das doenças relacionadas ao excesso de peso, além de possivelmente resultar em adultos menos produtivos e com pior qualidade de vida^6^.

A exposição excessiva à publicidade de alimentos pouco saudáveis é um dos fatores determinantes da obesidade infantil^7^, uma vez que pode influenciar as preferências alimentares das crianças, favorecendo o consumo dos alimentos anunciados. Isso ocorre pela dificuldade das crianças em reconhecer o caráter mercadológico das campanhas publicitárias, dadas suas limitações cognitivas^8^. Por esse motivo, organismos de saúde consideram a publicidade direcionada às crianças uma prática inapropriada e injusta, que precisa ser regulada^9^.

No Brasil, toda a publicidade direcionada ao público infantil é considerada abusiva e ilegal pelo Código de Defesa do Consumidor (CDC)^10^e pela Resolução nº 163/2014, do Conselho Nacional dos Direitos da Criança e do Adolescente (Conanda)^11^. Porém, dados de monitoramento da publicidade de alimentos apontaram que metade dos anúncios desse tipo veiculados em 2018, na programação de três canais abertos na televisão brasileira, possuíam conteúdos direcionados às crianças com mensagens encorajando o consumo excessivo de alimentos ultraprocessados^12^. Além disso, um recente estudo de monitoramento de páginas oficiais de empresas produtoras desse tipo de alimentos em plataformas de mídias sociais evidenciou o uso de estratégias de publicidade voltadas ao público infantil, tais como a presença de personagens infantis e a distribuição de brindes ou colecionáveis, sobretudo no YouTube^13^.

No ambiente digital, o YouTube é uma plataforma de compartilhamento de vídeos usada para diferentes tipos de comunicação, dentre eles a publicidade^14^. Crianças têm sido usuárias ativas dessa plataforma, tanto como espectadoras quanto compartilhando seu próprio conteúdo ( *youtubers mirins)* . Apesar do YouTube possuir restrição de cadastro para indivíduos menores de 13 anos, é possível acessar o conteúdo da plataforma sem a necessidade de inscrição. Além disso, existe uma plataforma específica de conteúdo para crianças, o YouTube Kids, que restringe a publicidade de anúncios pagos que promovam alimentos, independentemente de seu teor nutricional, porém, não há proibição sobre o conteúdo dos vídeos enviados por usuários que tenham uma conta ativa na plataforma, sendo possível que práticas publicitárias não sujeitas às políticas de publicidade da plataforma estejam presentes nos vídeos do YouTube Kids^15^.

Até o momento, o conteúdo de publicidade produzido por empresas de alimentos ultraprocessados em canais do YouTube no Brasil é conhecido^13^, mas não se sabe sobre a presença de publicidade de alimentos em canais do YouTube direcionados a crianças. Essa plataforma é popular entre crianças brasileiras e, segundo dados de pesquisas de monitoramento de mídia, 78% de uma amostra (n = 1.628) de pais de crianças entre 10 e 12 anos afirmam que seus filhos acessam o YouTube em seus *smartphones* ^16^. Soma-se a isso o elevado montante das receitas globais de publicidade do YouTube, que, apenas em 2020, totalizaram 19,77 bilhões de dólares^17^.

No meio digital, a criança pode interagir diretamente com o conteúdo publicitário por meio de curtidas, comentários e compartilhamentos, além de acessar links e jogos on-line^18^. Com isso, espera-se que o impacto da exposição de crianças à publicidade de alimentos sobre o seu comportamento alimentar seja ainda mais relevante nesse meio do que nas mídias tradicionais^19^. Diante disso, este artigo teve como objetivo analisar a publicidade de alimentos nos canais de YouTube direcionados à criança no Brasil em 2018 e a interação do público com essa publicidade.

## MÉTODOS

A amostra de canais do YouTube utilizada no estudo foi selecionada da lista dos 250 canais mais vistos no país, independentemente do público-alvo ou assunto abordado, segundo o portal SocialBlade – site de análise de mídia social independente do YouTube^a^–, em 23 de novembro de 2018. Cada canal dessa lista foi analisado individualmente, segundo a sua descrição e público-alvo indicado, e foram de interesse para o estudo os 25 primeiros colocados que apresentavam conteúdo direcionado a crianças ( Quadro 1 ). Para isso, adotaram-se os critérios da Resolução Conanda nº 163/2014^11^, que qualifica os elementos de uma comunicação mercadológica dirigida à criança, tais como a presença de: linguagem infantil, efeitos especiais e excesso de cores; trilhas sonoras de músicas infantis ou cantadas por vozes de criança; representação de crianças (por exemplo, *youtubers mirins)* ; pessoas ou celebridades com apelo ao público infantil; personagens ou apresentadores infantis; desenhos animados ou animação, e bonecos ou similares.

**Quadro 1:** Top 25 canais do YouTube de conteúdo infantil; ordenados por número total de visualizações.

Nome do canal	Data de criação	Ranking	Inscritos	Visualizações
Galinha Pintadinha	30/08/2006	2	14.313.531	10.169.800.278
Turma da Mônica	30/07/2012	4	9.328.729	7.267.014.998
Totoykids	02/12/2014	8	11.682.586	5.683.096.765
Erlaina e Valentina	13/04/2014	28	10.411.610	2.534.875.335
PatatiPatatá	15/08/2011	29	3.756.167	2.528.137.084
Paulinho e Toquinho	11/03/2015	31	4.294.953	2.381.444.545
Canal Kids HD	02/05/2017	32	5.524.762	2.328.708.524
Planeta das Gêmeas	20/11/2015	36	9.056.517	2.175.707.183
Fran, Nina e Bel para meninas	10/08/2013	39	6.663.106	1.942.418.375
Julia Minegirl	21/09/2015	41	3.435.392	1.924.067.618
O Reino das Crianças	19/08/2014	42	3.165.953	1.903.409.294
KidsFun	26/08/2015	43	8.528.323	1.854.220.383
Chiquititas SBT	22/05/2013	45	3.547.862	1.811.340.664
CartoonKIDS BR	21/10/2017	48	4.693.485	1.787.588.001
Bela Bagunça	27/03/2015	52	8.009.492	1.628.998.411
Juliana Baltar	09/05/2010	58	7.701.365	1.527.991.011
Maria Carla e JP	05/09/15	59	5.784.087	1.507.850.538
Clubinho da Laura	19/08/12	62	5.167.755	1.475.654.566
Xuxa VEVO	11/09/09	71	1.183.641	1.337.921.235
Fran para meninas	23/09/12	80	4.891.034	1.184.063.769
CanalKids- Tatá e Henrique	21/07/15	81	3.743.958	1.152.844.510
Canal da Lelê	25/08/14	85	3.819.222	1.125.696.017
Disney Channel Brasil	03/05/08	86	2.560.522	1.109.790.068
3 Palavrinhas	24/09/13	96	1.381.620	970.358.193

Os 10 vídeos mais populares de cada um dos 25 canais selecionados foram incluídos no estudo (n = 250). Esse número foi estabelecido com base na metodologia de um estudo similar realizado na Malásia^20^. No momento da coleta de dados, dois vídeos não estavam disponíveis para visualização devido à exclusão do conteúdo, totalizando uma amostra final de 248 vídeos.

Todos os vídeos foram assistidos de forma independente por duas pesquisadoras na plataforma do YouTube, usando o navegador Google Chrome, de janeiro a fevereiro de 2019. A programação dos vídeos totalizou 33 horas e 39 minutos. A data de publicação dos vídeos assim como os dados de número de visualizações e interações (“gostei” e “não gostei”) foram registrados e tabulados separadamente pelas pesquisadoras em planilhas do Excel. Além disso, elas identificaram se o canal era liderado por *youtubers mirins* ou não.

Para a coleta de dados de publicidade nos vídeos, considerou-se que no YouTube existem quatro tipos principais de anúncios: (a) anúncios ou vídeos de visualização não obrigatória, ou seja, é possível seguir para o vídeo de interesse sem visualizá-los, (b) anúncios ou vídeos de visualização obrigatória que, independentemente de sua duração, não possibilitam acessar o vídeo de interesse sem antes vê-los, (c) anúncios em formato de tarja que aparecem na parte superior ou inferior do vídeo e (d) marcas e produtos mencionados ou mostrados no cenário ou pelos personagens/apresentadores dos vídeos. Os três primeiros tipos de anúncios, (a), (b) e (c), podem aparecer em qualquer momento do vídeo, quando autorizados pelo proprietário do canal, que escolhe o formato e a frequência, mas não o conteúdo da publicidade. Esses anúncios foram excluídos deste estudo por não apresentarem um padrão de exibição, não tendo nenhuma relação com o conteúdo dos vídeos. Desse modo, foi considerado como publicidade somente as marcas mencionadas ou mostradas nos vídeos tipo (d). Nesse caso, a publicidade se deu pela representação da criança consumindo o produto da marca, pela demonstração de características do alimento da marca ou pela exibição da marca do alimento no plano de fundo do vídeo ( Quadro 2 ).

**Quadro 2:** Capturas de telas com exemplos de ocorrência da publicidade de alimentos em vídeos de YouTube direcionados às crianças no Brasil, 2018.

Tipo de publicidade	Contexto de aparição dos alimentos
Representação de indivíduo consumindo o produto da marca	*Uma criança consome um doce preparado com chocolates das marcas Hershey’s e M&M’s com a ajuda de um familiar.*
	*Uma criança consome um biscoito da marca Club Social enquanto navega pelo celular.*
Demonstração de características do alimento da marca	*Exibição de produtos da Coca-Cola e de balas da marca Mentos durante a execução de um experimento feito por uma criança e seu familiar.*
	*Abertura de um Kinder Ovo e exibição dos brinquedos que o acompanham.*
Exibição da marca do alimento no plano de fundo	*Exibição da logo da Pepsi no plano de fundo do vídeo.*
	*Exibição de produtos Cheetos no plano de fundo do vídeo.*

Todas as publicidades identificadas nos vídeos foram classificadas de acordo com o tipo de produto anunciado em: publicidade geral, a exemplo de brinquedos e jogos, e publicidade de alimentos e de serviços de alimentação. Quando identificada a presença de publicidade de alimentos no conteúdo do vídeo, foi efetuada a descrição dos produtos e de suas respectivas marcas. Esses primeiros foram agrupados em alimentos *in natura* e minimamente processados, ingredientes culinários, alimentos processados e ultraprocessados, segundo a classificação NOVA proposta por Monteiro et al.^21^No caso de publicidades de alimentos ultraprocessados, fez-se a classificação dos produtos em diferentes grupos, sendo esses: doces e guloseimas, bebidas lácteas, sucos e refrigerantes, pães e biscoitos de pacote, salgadinhos tipo *chips* , carnes ultraprocessadas e cereais matinais. Nos casos em que houve exibição ou menção de uma marca de alimentos no vídeo, sem que seu produto fosse exibido ou mencionado, considerou-se publicidade de alimentos e a classificação foi realizada segundo a predominância dos produtos dessa empresa. Para isso, procedeu-se à análise do portfólio de produtos nos sites oficiais das empresas. Em todos os casos, as empresas correspondentes às marcas encontradas nos vídeos foram identificadas.

Ao final, as planilhas de dados elaboradas pelas pesquisadoras do estudo foram comparadas e todas as incongruências corrigidas, a fim de garantir maior confiabilidade dos dados, cuja análise contemplou a descrição dos vídeos quanto ao número de visualizações e de interações do tipo “gostei” e “não gostei”. Para isso, utilizou-se a mediana e o intervalo interquartil (percentil 25; percentil 75), pelo fato da distribuição dessas variáveis não ter aderido à curva de distribuição normal, segundo o teste de normalidade Shapiro-Wilk. Além disso, calculou-se a frequência de publicidade geral, de alimentos (e suas subclassificações) e de serviços de alimentação, assim como o número de veiculações de publicidades de alimento em cada vídeo. Essa frequência foi estimada na amostra geral e nos vídeos de canais liderados ou não por *youtubers mirins* . Finalmente, realizou-se comparação das medianas do número de visualizações e de interações do tipo “gostei” e “não gostei” entre os vídeos que tiveram publicidade geral, de alimentos e de serviços de alimentação, por meio da aplicação do teste de Mann-Whitney, ao nível de significância de 5% (p < 0,05). As análises foram realizadas no software estatístico Stata, versão 12.0.

## RESULTADOS

A amostra foi composta por vídeos publicados entre os anos de 2006 e 2018, sendo a maior parte (83,2%) postada entre 2015 e 2018. Os vídeos apresentaram grande número de visualizações (mediana: 2,93 × 10^7^) e interações (mediana “gostei”: 1,09 × 10^5^; mediana “não gostei”: 0,25 × 10^5^). Do total de canais analisados, 9 (36%) eram de *youtubers mirins* (dado não apresentado em tabela).

A publicidade geral foi identificada em 45,6% (n = 113) dos vídeos, enquanto a publicidade de alimentos e de serviços de alimentação foi de 12,9% (n = 32) e 1,6% (n = 4) dos vídeos, respectivamente. Os anúncios publicitários de alimentos foram representados, em maior parte, pelos alimentos ultraprocessados (n = 30; 93,8%) e, dentre estes, por doces e guloseimas (n = 21; 70%). Em vídeos de canais liderados por *youtubers mirins,* houve maior frequência de publicidade geral (75,6% vs. 30,7%), de alimentos (24,4% vs. 7,2%) e de serviços de alimentação (3,7% vs. 0,6%). Ademais, as publicidades do subgrupo de alimentos ultraprocessados foram mais frequentes nos vídeos liderados por *youtubers mirins* , exceto as publicidades de sucos ou refrigerantes e cereais matinais – vídeos liderados por *youtubers mirins* : 16,7% e 0%, respectivamente, e vídeos não liderados por *youtubers mirins* 25% e 8,3%, respectivamente ( Tabela 1 ).

**Tabela 1:** Caracterização da publicidade presente em vídeos do YouTube direcionados à criança no Brasil, 2018.

Tipo de publicidade	Presença da publicidade^a^						Veiculações de publicidade de alimentos^b^		
	
	Amostra geral		Vídeos de canais de *youtubers mirins*				Amostra geral	Vídeos de canais de *youtubers mirins*	
	
			Sim		Não			Sim	Não
					
	n	%	n	%	n	%	n	n	n
Geral (exceto alimentação)	113	45,6	62	75,6	51	30,7	-	-	-
Alimentos	32	12,9	20	24,4	12	7,2	72	53	19
*In natura* /minimamente processados	2	6,3	2	10	0	0	3	3	0
Ingredientes culinários	2	6,3	2	10	0	0	2	2	0
Ultraprocessados	30	93,8	18	90	12	100	67	48	19
Doces e guloseimas	21	70	14	77,8	7	58,3	30	23	7
Bebidas lácteas	7	23,3	5	27,8	2	16,7	8	6	2
Pães e biscoitos de pacote	5	16,7	4	22,2	1	8,3	10	6	4
Sucos e refrigerantes	6	20	3	16,7	3	25	10	6	4
Salgadinhos tipo *chips*	3	10	2	11,1	1	8,3	7	6	1
Carnes ultraprocessadas	1	3,3	1	5,6	0	0	1	1	0
Cereais matinais	1	3,3	0	0	1	8,3	1	0	1
Serviços de alimentação	4	1,6	3	3,7	1	0,6	4	3	1

^a^Refere-se a presença de publicidade (Sim ou Não). Um mesmo anúncio pode conter publicidade geral, de alimentos e de serviços de alimentação, simultaneamente. Do mesmo modo, um anúncio pode ter mais de um tipo de alimento sendo anunciado.

^b^Refere-se ao total de vezes em que a publicidade de determinado tipo de produto foi notada nos vídeos avaliados. Aplica-se somente à publicidade de alimentos e serviços de alimentação.

Além disso, dos 30 vídeos que continham publicidade de alimentos ultraprocessados, 67 veiculações foram contabilizadas. As publicidades de doces e guloseimas (n = 30), sucos e refrigerantes (n = 10) e pães e biscoitos de pacote (n = 10) foram as mais incidentes. No caso dos vídeos de canais liderados por *youtubers mirins* , as veiculações de publicidade de alimentos foram 2,79 vezes mais frequentes do que nos vídeos de canais não liderados por *youtubers mirins* . Para a publicidade de alimentos ultraprocessados essa relação foi de 2,53 vezes ( Tabela 1 ).

Quanto às marcas evidenciadas/mencionadas nos vídeos, houve maior participação de empresas transnacionais do segmento de doces e guloseimas (18 marcas distintas) e produtoras de pães e biscoitos (nove marcas). As marcas mais frequentes são de empresas transnacionais como Pepsico, Mondelez e Nestlé ( Tabela 2 ).

**Tabela 2:** Empresas de alimentos e serviços de alimentação em publicidade de vídeos do YouTube direcionados à criança no Brasil, 2018.

Segmento da Empresa	n	Empresa (número de marcas)^a^
*In natura* /minimamente processados	2	Yoki (1); Fina (1)
Ingredientes culinários	1	Liza (1)
Ultraprocessados	45	
Doces e guloseimas	18	Mondelez (4); Nestlé (3); Ferrero (3); Chupa-Chups (1); Perfetti Van Melle (1); Garoto (1); Dori (1); Haribbo (1); Fini (1); Ovomaltine (1); Mars (1)
Bebidas lácteas	6	Danone (2); Nestlé (2); Sufresh (1); Pepsico (1)
Pães e biscoitos de pacote	9	Mondelez (2); Nestlé (2); Pandurata Alimentos (1); Parati (1); Pepsico (1); Richester (1); Wickbold (1)
Sucos e refrigerantes	5	Coca-Cola (3); Pepsico (1); Ative Plus (1)
Salgadinhos tipo *chips*	5	Pepsico (5)
Carnes ultraprocessadas	1	BRF (1)
Cereais matinais	1	Kelloggs (1)
Serviços de alimentação	4	McDonalds (1); Burger King (1); Hot Box (1); Los Paleteros (1)
Total	52	

^a^Uma mesma empresa pode ser comum a mais de uma marca na amostra.

O número de visualizações dos vídeos com publicidade geral foi menor do que daqueles que não a possuíam: 2,60 × 10^7^vs. 3,41 × 10^7^, p = 0,0391. Já o número de interações do tipo “gostei” foi maior entre os vídeos com publicidade geral (1,35 × 10^5^) em relação aos vídeos sem publicidade geral (0,84 × 10^5^), p = 0,0001 ( Tabela 3 ).

**Tabela 3:** Associação entre a presença de publicidade geral, de alimentos e de serviços de alimentação nos vídeos do YouTube direcionados à criança no Brasil com o número de visualizações e de engajamento dos usuários, 2018.

Tipo de publicidade	Visualizações		“Gostei”		“Não gostei”	
		
	Mediana	P25; P75	Mediana	P25; P75	Mediana	P25; P75
Geral						
Sim	2,60 × 10^7^	1,74 × 10^7^; 3,93 × 10^7^	1,35 × 10^5^	0,88 × 10^5^; 2,50 × 10^5^	0,29 × 10^5^	0,16 × 10^5^; 0,46 × 10^5^
Não	3,41 × 10^7^	1,47 × 10^7^; 7,30 × 10^7^	0,84 × 10^5^	0,54 × 10^5^; 1,75 × 10^5^	0,23 × 10^5^	0,14 × 10^5^; 0,47 × 10^5^
Valor p	0,0391		0,0001		0,2032	
Alimentos						
Sim	2,55 × 10^7^	1,75 × 10^7^; 3,92 × 10^7^	1,67 × 10^5^	1,04 × 10^5^; 3,27 × 10^5^	0,33 × 10^5^	0,23 × 10^5^; 0,39 × 10^5^
Não	3,07 × 10^7^	1,53 × 10^7^; 5,97 × 10^7^	1,02 × 10^5^	0,59 × 10^5^; 1,96 × 10^5^	0,24 × 10^5^	0,13 × 10^5^; 0,52 × 10^5^
Valor p	0,5938		0,0272		0,185	
Serviços de alimentação						
Sim	2,71 × 10^7^	2,57 × 10^7^; 3,84 × 10^7^	1,47 × 10^5^	1,37 × 10^5^; 1,97 × 10^5^	0,34 × 10^5^	0,33 × 10^5^; 0,35 × 10^5^
Não	2,96 × 10^7^	1,57 × 10^7^; 5,75 × 10^7^	1,06 × 10^5^	0,59 × 10^5^; 2,15 × 10^5^	0,24 × 10^5^	0,14 × 10^5^; 0,51 × 10^5^
Valor p	0,9824		0,2259		0,346	

No caso da publicidade de alimentos, do mesmo modo, o número de vezes que os vídeos foram marcados com “gostei” foi maior na presença deste tipo de publicidade (1,67 × 10^5^) em comparação aos vídeos sem publicidade de alimentos (1,02 × 10^5^), p = 0,0272 ( Tabela 3 ).

## DISCUSSÃO

O presente estudo evidenciou o fato de que a publicidade geral e a de alimentos está presente de forma expressiva nos canais brasileiros do YouTube direcionados ao público infantil, sobretudo em canais liderados por *youtubers mirins* . Além disso, foi identificado que a interação dos usuários pela marcação do tipo “gostei” é maior quando há anúncios publicitários, independentemente do tipo de produto que esteja sendo promovido.

Esses resultados denotam o elevado potencial de exposição de crianças brasileiras ao conteúdo publicitário em canais do YouTube no Brasil, sobretudo quando se trata de alimentos ultraprocessados de importantes empresas transnacionais. Esse cenário é crítico, por se tratar de mensagens publicitárias que se direcionam a um público reconhecidamente vulnerável^9^, que deveria estar sendo protegido desse tipo de mensagem. Essa exposição ocorre em um meio (YouTube) de grande popularidade, que pode ser acessado por crianças a qualquer momento por diferentes dispositivos, sem a supervisão direta de seus pais ou responsáveis. Soma-se a isso a conhecida associação entre exposição à publicidade de alimentos e preferência pelos produtos anunciados^8^e entre o consumo de alimentos ultraprocessados e desfechos desfavoráveis na saúde de crianças e adolescentes, incluindo a obesidade^4^.

Os resultados do estudo também evidenciam que, além das crianças serem audiência do conteúdo publicizado, elas também são criadoras de conteúdo, como no caso dos *youtubers mirins* . Nos vídeos produzidos por essas crianças, houve maior frequência e maior número de veiculações de publicidade de alimentos. O conteúdo dos vídeos de *youtubers mirins* contém integrações sutis entre publicidade de marcas e entretenimento, o que os torna mais difíceis de serem reconhecidos como conteúdo publicitário^22^. Além disso, quando um conteúdo comercial é endossado por uma criança, esta se torna importante influenciadora para as decisões de consumo do seu público, que em geral costuma ser constituído de outras crianças^22^.

A taxa de publicidade de alimentos ultraprocessados identificada neste estudo é preocupante. Resultados semelhantes foram encontrados na Malásia, onde 56,3% da publicidade de alimentos veiculada em canais infantis do YouTube eram de alimentos ultraprocessados, principalmente de *fast food* e doces, resultando na transmissão de 0,73 anúncios a cada hora de vídeo^20^. No Reino Unido, 92,9% dos vídeos de influenciadores de crianças no YouTube sugeriam algum alimento ou bebida^23^, enquanto na Espanha essa taxa foi de 73,6%^14^.

Além da presença de publicidade de alimentos nos canais do YouTube direcionados à criança no Brasil, uma expressiva interação com o conteúdo dos vídeos foi identificada, na presença do conteúdo publicitário, pela marcação “gostei”. Dados na literatura apontam que, quanto maior a interação das crianças com a publicidade, maior é o efeito da exposição no comportamento alimentar^19^. Na Austrália, por exemplo, um estudo mostrou que adolescentes de 12 a 17 anos que curtiram ou compartilharam uma postagem de alimento ou bebida em mídias sociais pelo menos uma vez no mês anterior à pesquisa tiveram alta ingestão de alimentos não saudáveis, e quanto mais os adolescentes interagiam com o conteúdo, maior era o consumo^24^.

Em conjunto, as evidências encontradas no presente estudo caracterizam a baixa efetividade das medidas de regulação de publicidades direcionadas às crianças no Brasil, que são consideradas abusivas e proibidas por lei^10 , 11^. Cabe, portanto, avançar nessa agenda, o que implica atualizar os termos regulatórios no contexto da publicidade digital e garantir a fiscalização e penalização para esse tipo de publicidade.

No primeiro caso, é necessário acrescentar aos textos regulatórios a definição clara de publicidade no meio digital, inclusive daquela realizada por influenciadores digitais (como *youtubers mirins* )^25^. Alguns textos regulatórios que podem ser usados como inspiração à regulação da publicidade digital de alimentos no Brasil é a Lei nº 30/2019, de Portugal, que introduz restrições à publicidade de alimentos não saudáveis na internet, em sites ou mídias sociais, bem como em aplicativos móveis, quando seu conteúdo tem como destinatários crianças abaixo de 16 anos^26^. Na América Latina, o governo chileno também restringe a publicidade de alimentos considerados não saudáveis direcionada a crianças menores de 14 anos em sites ^27^ .

Além disso, recomenda-se a imposição de limites para o direcionamento da publicidade por meio de algoritmos de dados individuais^25^, pois atualmente as empresas podem extrair dados de localização e histórico de navegação do usuário de um dispositivo digital e usá-los para personalizar as mensagens publicitárias. Esse é um processo automatizado, denominado mídia programática, usado na compra e entrega de anúncios por meio de algoritmos para encontrar e segmentar a publicidade segundo os interesses dos indivíduos. Assim, os anunciantes podem identificar onde a criança está e seus conteúdos preferidos. Com isso, as empresas podem experimentar diferentes estratégias comerciais direcionadas ao perfil de consumo do indivíduo e monitorar a interação deste com o anúncio, assim como a conversão em aquisição^18^.

No caso da fiscalização, é necessário investir em tecnologias para o monitoramento sistemático da publicidade digital, que capturem a multidimensionalidade desse tipo de mídia e as diferentes formas de se fazer publicidade^19^. Um caminho seria a adoção de instrumentos predefinidos para a identificação da publicidade de alimentos direcionada às crianças em canais do YouTube, inclusive em canais liderados por influenciadores infantis, conforme previsto nos protocolos lançados recentemente pelo escritório regional da Organização Mundial da Saúde na Europa^b^. Deve-se, ainda, superar os desafios para o monitoramento da publicidade disseminada pela mídia programática, não abordada no presente estudo, nos tipos de publicidade que antecedem ou são veiculados ao decorrer dos vídeos, geralmente de visualização obrigatória, mas que não têm um padrão de exibição igual para os indivíduos.

As plataformas de mídias sociais também podem contribuir na garantia da proteção de crianças à exposição de conteúdo comercial. No caso do YouTube, conforme já mencionado, não há qualquer restrição à publicidade infantil, ainda que seja possível o acesso de crianças à plataforma. Quanto ao YouTube Kids, apesar de haver proibição à publicidade de alimentos realizada pelas grandes empresas, essa medida não se estende ao conteúdo gerado por influenciadores e há evidências de grande inadequação e negligência por parte da plataforma na fiscalização ^28^ .

Do mesmo modo, a indústria de alimentos pode atuar de forma responsável e assumir o compromisso de não se comunicar diretamente com crianças. No Brasil, o Conselho Nacional de Autorregulamentação Publicitária (Conar), composto por agências de publicidade e anunciantes, não reconhece a publicidade direcionada às crianças como ilegal e apresenta uma atuação mais alinhada às relações de mercado do que voltadas à proteção de crianças em face do poder das práticas publicitárias ^29^ .

Cabe discutir o precedente jurídico que multou uma empresa de alimentos ultraprocessados no Brasil, a Pandurata Alimentos, por direcionar publicidade às crianças. Apesar de não ser um caso de publicidade disseminada no meio digital, foco deste estudo, o Superior Tribunal de Justiça (STJ) reconheceu que a empresa usava de estratégia promocional para incentivar o consumo de alimentos de baixa qualidade nutricional por crianças e, assim, contrariava as diretrizes sobre abusividade do CDC^30^. Ampliar o número de precedentes jurídicos a respeito de publicidade abusiva e expandir o reconhecimento de que também é abusiva a contratação de *youtubers mirins* para publicidade de produtos são caminhos necessários para garantir a proteção de crianças contra a comunicação mercadológica.

Quanto aos potenciais limites do estudo, indica-se primeiramente a ausência de um padrão de exibição dos anúncios de visualização obrigatória, opcional e em tarjas, o que permitiu avaliar apenas uma parcela da publicidade de alimentos em vídeos voltados para o público infantil. Com isso, estima-se que a exposição de crianças ao conteúdo publicitário seja ainda maior nessa plataforma. Em segundo lugar está a impossibilidade de saber a origem das interações, ou seja, quantas delas vieram de fato do público infantil.

Apesar disso, foi possível concluir que há predomínio da publicidade de alimentos ultraprocessados em vídeos de canais infantis do YouTube no Brasil, sobretudo em canais liderados por *youtubers mirins* , o que reforça a importância de ações de regulamentação da publicidade de alimentos nas mídias sociais e fornece evidências para futuras investigações que visem compreender o impacto da exposição infantil à publicidade de alimentos e o perfil de consumo alimentar das crianças.

## CONCLUSÃO

Foi evidenciada expressiva presença de publicidade de alimentos de importantes transnacionais, com alta frequência de veiculações em canais infantis no YouTube, sobretudo em canais liderados por *youtubers mirins* . Ademais, houve mais interações do tipo “gostei” em vídeos com publicidade geral ou de alimentos.

## Footnotes

a Social Blade. Top 100 youtubers in Brazil sorted by SB rank [internet]. 2018 [citado 23 nov 2018]. Disponível em: https://socialblade.com/youtube/top/country/br

b World Health Organization. Europe. Monitoring of marketing of unhealthy products to children and adolescents:– protocols and templates. 2020 [citado 20 ago 2021]. Disponível em: https://www.euro.who.int/en/health-topics/disease-prevention/nutrition/activities/monitoring-of-marketing-of-unhealthy-products-to-children-and-adolescents-protocols-and-templates
